# Treating Allergic Bronchopulmonary Aspergillosis: A Review

**DOI:** 10.7759/cureus.4538

**Published:** 2019-04-24

**Authors:** Avani R Patel, Amar R Patel, Shivank Singh, Shantanu Singh, Imran Khawaja

**Affiliations:** 1 Internal Medicine, Northern California Kaiser Permanente, Fremont, USA; 2 Internal Medicine, Southern Medical University, Guangzhou, CHN; 3 Pulmonary Medicine, Marshall University School of Medicine, Huntington, USA

**Keywords:** allergic bronchopulmonary aspergillosis, itraconazole, asthma, hypersensitivity reaction, aspergillus fumigatus, pulmonary fibrosis, prednisolone, omalizumab, voriconazole

## Abstract

Allergic bronchopulmonary aspergillosis (ABPA) is a pulmonary disorder that results from a hypersensitivity reaction to the fungi Aspergillus fumigatus (Af). It presents with pulmonary infiltrates and bronchiectasis. Past research studies on ABPA have led to the conclusion that it is both underdiagnosed and much more prevalent than previously assumed. The underdiagnosing of ABPA is due to a lack of consensus regarding diagnosis and treatment. Complications that result from delay in treatment for ABPA are pulmonary fibrosis, bronchiectasis with chronic sputum production, and severe persistent asthma with loss of lung function. Because of this, it becomes imperative that ABPA treatment guidelines are reviewed and more thoroughly evaluated regarding their efficacy. The following article addresses the epidemiology, the pathophysiology, and the treatment of ABPA. The treatment is studied in detail regarding the types of medications used and their proven clinical impact on patients according to past research studies. The aim of this article is to address the current need for larger clinical trials in order to learn more and establish more formal treatment protocols for ABPA.

## Introduction and background

Dr. K.F. Hinson first described allergic bronchopulmonary aspergillosis (ABPA) in 1952 [1]. Several years later in 1968 ABPA was first reported in the United States [2]. This caused an increased awareness of ABPA on a global scale. Greenberger and Patterson studied 531 asthmatic patients from the United States between 1983 and 1986 [3]. They found that 32 patients or 6% had immediate cutaneous reactivity to Aspergillus antigens, the causative antigen in ABPA. ABPA is an immune-related pulmonary disorder that results from hypersensitivity to the Aspergillus fumigatus (Af) fungi. It is seen more commonly in patients with asthma or cystic fibrosis. In susceptible hosts, a hypersensitivity reaction is evoked by repeated inhalation of Aspergillus spores [4]. These Aspergillus spores become trapped in the thick sputum of patients causing a cascade of inflammatory reactions that can result in ABPA [4]. ABPA can also be further classified according to the criteria that the patient meets (see Table 1). Serologic ABPA or ABPA-seropositive (ABPA-S) are asthma patients who meet the minimum criteria but do not have central or peripheral bronchiectasis [5-7]. ABPA-central bronchiectasis (ABPA-CB) are patients who meet the minimum criteria and also have central bronchiectasis [5-7]. Finally, severe asthma with fungal sensitivity (SAFS) are patients who have severe asthma and sensitivity to fungi but do not meet the criteria for ABPA [5-7]. Current studies on ABPA have established that ABPA is both underdiagnosed and much more prevalent than previously assumed [8]. At present, ABPA has an overall global prevalence of 2.5% [9]. The underdiagnosing of ABPA is due to lack of a standardized diagnostic criteria [4]. This leads to delay in treatment for ABPA patients, which can further lead to major complications like pulmonary fibrosis, bronchiectasis with chronic sputum production, and severe persistent asthma with loss of lung function. Research studies over the years have addressed different treatment strategies for ABPA but currently there is a lack of information regarding a standard treatment plan, the appropriate duration of treatment, dosages, and which medications to use. The following review article addresses the epidemiology, the pathophysiology, and the treatment of ABPA. It goes into detail regarding different medications used and their impact on patients as studied by past clinical trials. The aim of this article is to bring awareness regarding the lack of clinical research data on ABPA treatment and stress that more is needed to address current clinical needs (see Table 2).

**Table 1:** Subclassifications of ABPA ABPA: allergic bronchopulmonary aspergillosis; ABPA-S: ABPA-seropositive; ABPA-CB: ABPA-central bronchiectasis; SAFS: severe asthma associated with fungal sensitivity.

Classification	ABPA-S	ABPA-CB	SAFS
Findings	Patients with asthma that meet minimum requirements of ABPA but do not have central or peripheral bronchiectasis [5-7].	Patients who meet the minimum criteria for ABPA and also have central bronchiectasis [5-7].	Patients who have severe asthma and sensitivity to fungi but do not meet the criteria for ABPA [5-7].

**Table 2:** Recommendations for Diagnosing or Excluding ABPA in Every Asthma Patient Using Sequential Testing, Including How ABPA-S and ABPA-CB are Diagnosed ABPA: allergic bronchopulmonary aspergillosis; Af: aspergillus fumigatus; IgE: immunoglobulin E; IgG: immunoglobulin G, ABPA-CB: ABPA-central bronchiectasis; ABPA-S: ABPA-serologic.

Test	Result	Conclusion
Cutaneous (prick) test for Af [10]		
	Positive	Serologic study required
	Negative	Intradermal test required
Intradermal test for Af [10]		
	Positive	Serologic study required
	Negative	ABPA excluded
Serologic studies [10]		
Total serum IgE, ng/mL, Precipitins for Af (5 x concentrated serum) [10]	>2,000 Positive	Further serologic studies required
Total serum IgE precipitins [10]	<1,000 Negative	ABPA probably excluded (serologic studies should be repeated if chest roentgenographic infiltrates are found, even transiently)
Further serologic studies [10]		
IgE and IgG indexes [10]	Both <2	Not consistent with ABPA (indexes should be repeated if chest roentgenographic infiltrates are found, even transiently)
IgE and IgG indexes [10]	Both >2	ABPA diagnosed, tomography required for further evaluation
Chest tomography [10]		
	Central bronchiectasis seen	Diagnosis: ABPA-CB
	Normal	Diagnosis: ABPA-S

## Review

Epidemiology

Due to lack of data it was previously estimated that over four million people are affected by ABPA worldwide [11]. Currently it is believed that the actual global burden of ABPA exceeds 4.8 million [11]. By WHO region, the ABPA burden estimates are Europe, 1,062,000 (ranging from 297,000-1,487,000); the Americas, 1,461,000 (ranging from 409,000-2,045,000); Eastern Mediterranean, 351,000 (ranging from 98,000-491,000); Africa, 419,000 (ranging from 117,000-587,000); Western Pacific, 823,000 (ranging from 230,000-1,152,000); South East Asia, 720,000 (range 202,000-1,009,000) [11]. Worldwide, about 4,837,000 active asthma patients out of 193 million will develop ABPA, which has a prevalence rate of 2.51% [11].

Pathophysiology

**Figure 1:**
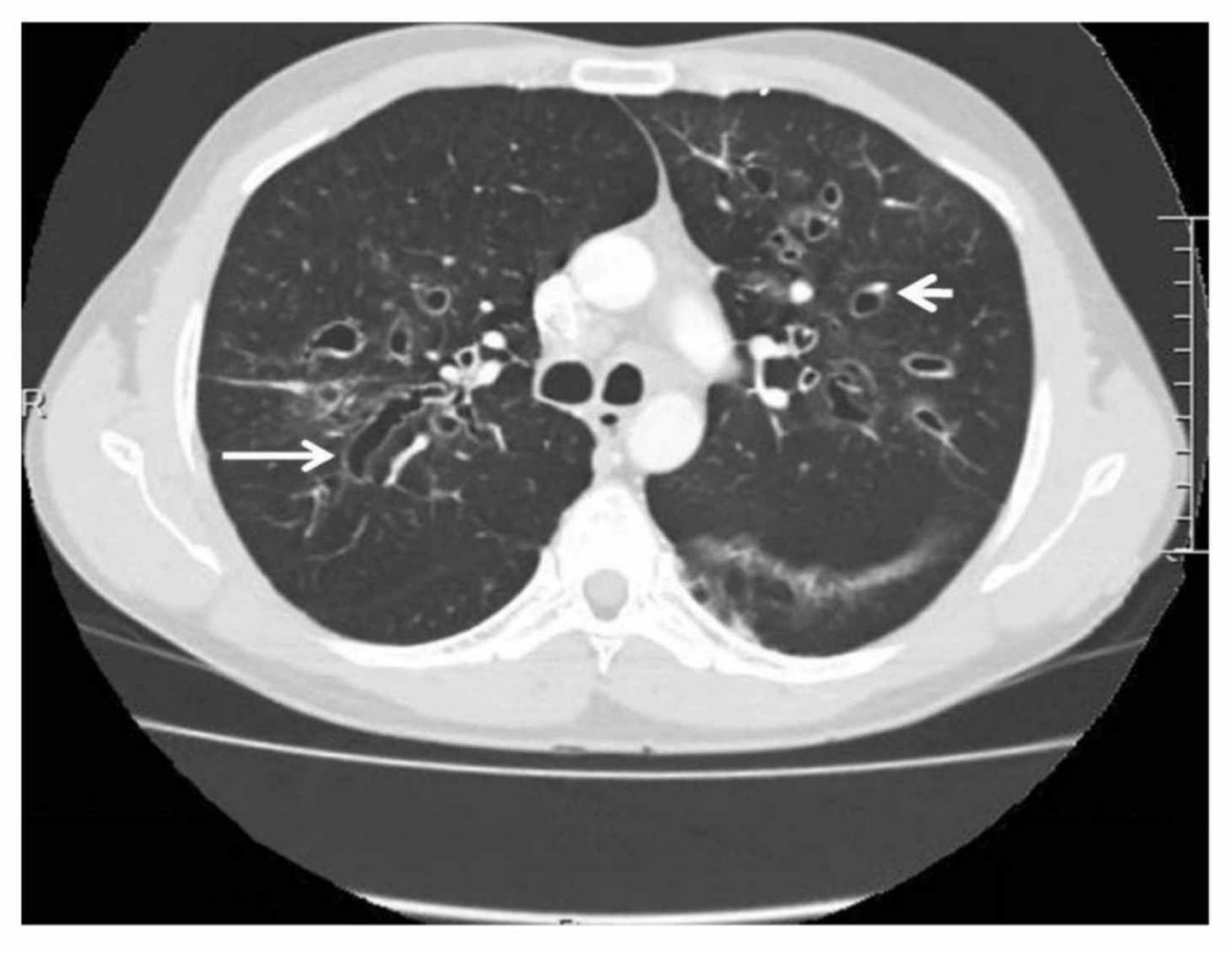
Computed Tomography Scan of Thorax Showing Central Bronchiectasis in Case of ABPA This is a computed tomography (CT) scan of the thorax showing central bronchiectasis [11]. It is identified by the 'signet ring' (short, thick arrow) and 'string of pearls' (long, thin arrow) appearances [11]. Mucoid impaction and dilated bronchi are also present [11].

ABPA is caused by a hypersensitivity reaction to Af antigens. In susceptible hosts like those with asthma or cystic fibrosis, this is caused by repeated inhalation of Aspergillus fumigatus spores. The hypersensitivity reaction elicited is mainly type I otherwise known as an immunoglobulin E (IgE) mediated reaction [12]. Both type III and type IV hypersensitivity reactions have also been seen but less commonly [12]. When the spores are inhaled, the Af antigens cause a polyclonal antibody response leading to elevated levels of total IgE, Af-IgE, and Af-IgG antibodies [13, 14]. Patients with expression of HLA-DR2 and HLA-DR5 genotypes are at risk for ABPA, while HLA-DQ2 is protective against ABPA [15, 16]. There are five stages of ABPA: (i) acute (Figure 1), (ii) remission, (iii) exacerbation, (iv) corticosteroid-dependent asthma, (v) fibrotic lung disease (see Table 3). Till date there is no standardized diagnostic criteria for ABPA, although many have been proposed over the years (see Table 4). More studies will be needed to formulate one.

**Table 3:** ABPA Staging, Radiographic Findings, and Corresponding IgE Levels ABPA: allergic bronchopulmonary aspergillosis; IgE: immunoglobulin E; CT: computed tomography.

Stage	Description	Radiographic Findings	Total IgE Concentration
Stage I: acute	The patient is diagnosed with ABPA. Some features such as Aspergillus-specific IgE, radiological abnormalities, peripheral blood eosinophilia, and Aspergillus-specific serum precipitins may be seen [12].	There may be homogenous infiltrates, mucus plugging, lobar consolidation or collapse, “tree-in-bud” appearance, bronchiectasis (see Figure 1) [8].	Overall elevated [8]
Stage II: remission	Asymptomatic patient with underlying controlled asthma but no new radiological infiltrates and no rise in total IgE for a minimum of six months [12].	No infiltrates are seen [8].	Normal or elevated IgE level but less than stage I level [8].
Stage III: exacerbation	New pulmonary infiltrates appear on x-ray with peripheral blood eosinophilia and double the remission level IgE levels [12].	The same findings as seen in acute stage [8].	Elevated IgE levels usually double the level of stage II [8].
Stage IV: steroid-dependent asthma	Patients become dependent on corticosteroid treatment and are unable to completely taper off from it [12].	No infiltrates are seen. There can be atelectasis or hyperinflation from asthma [8]. If exacerbation occurs then the findings will resemble stage I [8].	Normal or elevated IgE level [8].
Stage V: end-stage fibrotic disease	Chest x-ray and CT scans will show irreversible fibrosis and chronic cavitation. Despite this, serological parameters are usually negative [12].	There is lung scarring, hyperinflation, chronic infiltrates, fibrosis or cavities or fibrocavitary findings [8].	Normal or elevated IgE level [8].

**Table 4:** Diagnostic Criteria for ABPA ABPA: allergic bronchopulmonary aspergillosis; Af: Aspergillus fumigatus; CB: central bronchiectasis; CF: cystic fibrosis; IgE: immunoglobulin E; IgG: immunoglobulin G; ISHAM: International Society for Human and Animal Mycology.

1977, Rosenberg-Patterson criteria [17, 18]	2013, Truly Minimal Criteria [4]	2013, ISHAM Working Group [9]
Major Criteria	Criteria	Predisposing Conditions
(1) Asthma, (2) Presence of fleeting or fixed pulmonary opacities on chest radiograph, (3) Immediate cutaneous hypersensitivity reaction to Af, (4) Total serum IgE elevated, more than 1000 IU/mL, (5) Precipitating antibodies against Af, (6) Peripheral blood eosinophilia, (7) Central or proximal bronchiectasis with normal tapering of distal bronchi	(1) Asthma, (2) Immediate cutaneous hypersensitivity reaction to Af, (3) Total serum IgE elevated more than 1000 ng/mL (417kU/L), (4) CB in absence of distal bronchiectasis	(1) Asthma, (2) CF
		Obligatory Criteria (both need to be present)	
		(1) Type 1 Aspergillus skin test positive (immediate cutaneous hypersensitivity reaction to Af) or elevated IgE levels against Af, (2) Elevated total IgE levels more than 1,000 IU/mL (unless all other criteria is met, then total IgE levels can be less than 1,000 IU/mL)	
		Other criteria (two out of three at least)	
		(1) Presence of IgG antibodies against Af or precipitating antibodies, (2) Presence of fleeting or radiograph pulmonary opacities consistent with ABPA, (3) Eosinophil count more than 500 cells/μL in steroid naïve patient (may be a historical value)	
Minor Criteria			
(1) Golden brown sputum plugs in expectorant, (2) Positive sputum culture for Aspergillus species, (3) Late (arthus-type) skin reactivity to Af			

Treatment

There are four goals of treatment in ABPA. They are to control symp­toms of asthma or cystic fibrosis, to prevent or treat pulmonary exacerba­tions of ABPA, to reduce or remit pulmonary inflammation, and to reduce progression to end-stage fibrotic or cavitary disease [8]. As mentioned earlier, a delay in treatment for ABPA can lead to complications like pulmonary fibrosis, bronchiectasis with chronic sputum production, and severe persistent asthma with loss of lung function. ABPA is further classified into ABPA-S, ABPA-CB, or SAFS based on patients meeting or not meeting the minimum criteria for ABPA and their chest radiograph findings [10]. Despite the classification of the patient, the treatment doesn't change significantly. Corticosteroids still remain the main drug therapy used for ABPA regardless of classification.

Corticosteroids

Currently, systemic glucocorticoids remain the most effective drugs for treating ABPA [19]. Despite its therapeutic effectiveness, the optimal dosing schedule for prednisolone is currently not known and this is because of a lack of clinical trials [20-22]. The commonly used treatment strategy is an initial dose of prednisolone 0.5 mg/kg daily for 14 days, followed by 0.5 mg/kg every other day, and then further tapered and finally discontinued at three months [20].

An unblinded randomized clinical trial was done with 92 patients in whom the previously mentioned treatment strategy was compared to prednisolone 0.75 mg/kg/day for six weeks followed by a more gradual taper [22]. No significant difference was noted except that the rate of adverse effects was higher in the 0.75 mg/kg/day prednisolone group [22]. Response to prednisolone treatment was demonstrated by the subsequent reduction in serum IgE levels. Serum IgE levels should have a decrease of 25% after one month of treatment and about 60% after two months [23]. A total serum IgE level decrease of 35% is considered a good therapeutic response.

For acute ABPA, systemic glucocorticoids are the mainstay of treatment. This is based on the results of case series done on the topic. In one case series all 126 newly diagnosed ABPA patients responded to a course of oral glucocorticoids and developed remission (as demonstrated by decline of IgE levels below 35% and the chest becoming clear of radiographic infiltrates) [22]. A second case series with 84 ABPA patients had a cumulative response of the chest clearing of radiographic infiltrates, a 50% and over decline in total IgE, and return to normal levels of blood eosinophil counts [23]. All occurred in response to a course of prednisolone [23].

Antifungal Therapy

At present, only itraconazole and voriconazole are used in the treatment against ABPA and only for patients who are unable to taper oral prednisolone or have an ABPA exacerbation. When an antifungal agent is used, it is only used for 16 weeks. Due to lack of data and findings of current data, 16 weeks is considered the maximum time for an APBA patient to use antifungals like azoles with a therapeutic benefit.

One randomized clinical trial had 55 patients already on glucocorticoids receiving either itraconazole or a placebo for their corticosteroid-dependent ABPA [24]. There were responses in 13 of the 28 itraconazole patients, as compared with five of 27 patients in the placebo group [24]. A response was defined by the trial as a reduction of at least 50% in the glucocorticoid dose, a 25% decrease in the serum IgE levels, and one of the following: minimally 25% improvement in exercise tolerance or pulmonary-function tests, or a partial or complete resolution of pulmonary infiltrates [24].

In a second randomized clinical trial, 131 patients with both asthma and acute ABPA were divided into two groups in order to compare the therapeutic benefit of itraconazole 200 mg twice a day to prednisolone treatment [25]. The prednisolone treatment was 0.5 mg/kg/day for four weeks, followed by 0.25 mg/kg/day for four weeks, and then slowly tapered and discontinued at four months [25]. The itraconazole was also given for four months [25]. The number of subjects exhibiting a composite response was significantly higher in the prednisolone group (100% of patients) as compared with the itraconazole group (88% of patients) [25].

Itraconazole and voriconazole work by reducing the fungal load, which helps control the antigenic stimulus, and thus decrease the inflammatory response [26]. Itraconazole also has an added benefit in corticosteroid-dependent ABPA patients because of its ability to impair metabolism of glucocorticoids, thus raising plasma levels. This effect is seen more with methylprednisolone versus prednisolone. Past case reports have also noted development of Cushing’s syndrome in those types of patients [27-29]. Voriconazole as compared to itraconazole has better gastrointestinal tolerance and bioavailability but skin cancer has been associated with long-term use [30]. Recently, posaconazole use has also been discussed for ABPA treatment. A case series was done in 2012, where 33 courses of therapy were analyzed [31]. In that, 24 were with voriconazole and nine were with a newer drug, posaconazole [31]. A clinical response to voriconazole was observed in 17/24 (70%) patients at three months, 15/20 (75%) at six months, and 12/16 (75%) at 12 months compared with 7/9 (78%) at three, six, and 12 months for posaconazole [31]. Despite this, posaconazole is still not recommended for treatment because there have not been enough prospective studies done to evaluate posaconazole as an effective treatment strategy [31].

Omalizumab

Omalizumab is a humanized monoclonal antibody that works against IgE. Past studies have suggested that omalizumab may be used in the treatment of ABPA, especially in patients with asthma. Despite this, the use of omalizumab in ABPA patients with cystic fibrosis requires more definitive clinical trials.

A randomized placebo controlled trial was done to assess the clinical and immunologic effects of omalizumab in ABPA [32]. Thirteen patients with chronic ABPA were randomized to a four month treatment with omalizumab (750 mg monthly) or a placebo followed by a three-month washout period in a cross-over design [32]. The endpoint was the number of exacerbations. During the active treatment phases, the ABPA exacerbations were significantly less frequent than with omalizumab (two versus 12 events) [32].

In another open-label study, 16 ABPA patients without cystic fibrosis took omalizumab for one year. The omalizumab use was associated with a marked decrease in the number of asthma exacerbations and in needed oral glucocorticoid dose as compared with the year prior [33].

Monitoring of Treatment

The clinical response to glucocorticoid treatment should be monitored with regular measurements of the serum total IgE concentration every one to two months [34]. There should be a resolution of radiographic opacities and a 35% minimum reduction in serum total IgE levels [35]. Patients with a baseline of IgE less than 2500 IU/mL may have less than a 35% reduction [36]. If the serum total IgE levels double at any point then it is an indicator of ABPA exacerbation [23].

Treatment of ABPA-S

The treatment of ABPA-S is prednisone 0.5 mg/kg every day for 14 days, then on alternate days for three months, and then discontinued [23]. It may be continued if prednisone is required to control the asthma [23]. Total serum IgE needs to be measured every two months and baseline levels should be determined prior to starting prednisone therapy [23]. Other recommendations include continuing ambulatory management for control of asthma, for detection and therapy of ABPA exacerbations expressed by doubling of IgE and chest radiograph infiltrate [23].

Treatment of ABPA-CB

The treatment of ABPA-CB is very similar to ABPA-S. It is prednisone 0.5 mg/kg every day for 14 days, then on alternate days for three months, and then discontinued [23]. It may be continued if prednisone is required to control the asthma [23]. The total serum IgE needs to be measured every two months and baseline levels should be determined prior to starting prednisone therapy [23]. Other recommendations include classification as ABPA stage II (remission) through stage IV (steroid-dependent asthma) during subsequent ambulatory care period [23].

Treatment of ABPA in Cystic Fibrosis Patients

The treatment prescribed for ABPA in patients with cystic fibrosis is similar to the above mentioned regimens although there is a lack of research done. Systemic glucocorticoids are usually prescribed and result in a reduction of serum total IgE levels.

## Conclusions

The material reviewed in this paper focuses on the treatment of ABPA. It goes into detail regarding the different medications used and their demonstrated therapeutic benefit in treating ABPA. Despite these key points being addressed, larger and more prospective studies are needed to create a more standardized treatment regimen. It also needs to be evaluated whether the drugs should be given alternatively or as a combination for a more therapeutic effect. This is a review article for busy physicians and medical students to have a cumulative view of our current situation regarding the treatment of ABPA.
